# Infection Characteristics, Transcriptomics, and Metabolomics of African Swine Fever Virus SY-1 Strain in Orally Infected Weaned Landrace Piglets

**DOI:** 10.1155/tbed/2453420

**Published:** 2025-07-14

**Authors:** Jingyu Yang, Xiaotong Hu, Changjie Lv, Chuxing Cheng, Qiang Zhang, Xiaomei Sun, Xuezhu Du, Meilin Jin

**Affiliations:** ^1^State Key Laboratory of Agricultural Microbiology, Huazhong Agricultural University, Wuhan 430070, China; ^2^College of Veterinary Medicine, Huazhong Agricultural University, Wuhan 430070, China; ^3^State Key Laboratory of Biocatalysis and Enzyme Engineering, School of Life Sciences, Hubei University, Wuhan 430062, China; ^4^Hubei Jiangxia Laboratory, Wuhan 430200, China

**Keywords:** African swine fever virus, amino acid, antiviral effect, metabolomics, transcriptomics

## Abstract

African swine fever (ASF) is an acute infectious disease that significantly threats the global pig farming industry. At present, there is no efficient vaccine or targeted therapy for this virus, primarily because of the unclear pathogenesis of ASF virus (ASFV) infection and its interactions with host responses. Here, we established an oral infection model of ASFV in Landrace pigs and identified gene expression and metabolic changes in the pig spleen following ASFV infection using transcriptomic and metabolomic analyses. After ASFV SY-1 infection, 5556 differentially expressed genes (DEGs) were identified, wherein 2577 and 2979 were upregulated and downregulated, respectively. Gene Ontology (GO) and Kyoto Encyclopedia of Genes and Genomes (KEGG) analyses revealed that these genes were dynamically enriched in various biological processes, including the innate immune response, inflammatory response, chemokine signaling, and signal transduction. Integrated transcriptome and metabolome analyses indicated that ASFV altered diverse pathways, including cysteine and methionine metabolism, cGMP-PKG signaling, choline metabolism in cancer, cholesterol metabolism, sphingolipid signaling, protein digestion and absorption, FoxO signaling, and central carbon metabolism in cancer. Additionally, we confirmed that metabolites, such as L-glutamate, glycerophosphocholine, and L-serine, significantly inhibit ASFV proliferation in vitro. This study improves our comprehension of the relationships between viruses and hosts, and it serves as a guide for identifying new inhibitors for ASFV.

## 1. Introduction

African swine fever (ASF) is an acute infectious disease caused by the ASF virus (ASFV). Highly virulent strains have staggering morbidity and mortality rates of up to 100% among domestic pigs, seriously threating the pig industry worldwide. ASFV is classified under the Asfaviridae family and is the only known DNA arbovirus [1]. The ASFV genome consists of double-stranded DNA, ranging from 170 to 190 kb in size, which codes for 150–200 viral proteins [2]. Since its discovery in Kenya in 1921 [3], the ASFV has undergone two cross-continental transmissions, igniting outbreaks in numerous European countries, including Spain, Italy, Portugal, Georgia, and Russia [4, 5]. On August 3, 2018, ASF made its way into China, swiftly spreading across the country within months and subsequently invading other Asian nations, including Vietnam, Mongolia, and Cambodia [6, 7]. Currently, ASF has been reported in 80 countries worldwide, inflicting direct economic losses of over 100 billion US dollars on the global pig farming industry.

Extensive research has been conducted to develop vaccines and therapeutics for ASFV, and many significant advancements have been achieved [8–12]. However, due to the lack of commercially available vaccines or drugs, prevention and control currently rely mainly on comprehensive culling and stringent biosecurity measures [13]. This scenario mainly stems from a constrained comprehension of the pathogenic mechanisms involved in ASFV infection and the dynamics of its interactions with host cells.

Transcriptome analysis is a valuable tool for elucidating the relationship between genotypes and phenotypes, thereby enhancing our understanding of the underlying pathways and mechanisms governing cell fate, development, and disease progression [14]. Previous studies utilizing transcriptomic and single-cell transcriptomic approaches have revealed the substantial impact of ASFV infection on the host's innate immune response and metabolic pathways at the cellular and organism levels [15–18]. Moreover, the generation of small molecule metabolites and their fluctuations can directly reflect the outcomes of virus-regulated biological processes, prompting an increasing focus on the role of metabolites in disease progression [19–21]. Thus, metabolomics can help reveal changes in molecules at different physiological or pathological states, identify biomarkers, and elucidate the pathogenesis of diseases and potential therapeutic targets [22]. A metabolomic analysis of the spleens of hosts infected with ASFV indicates that ASFV can boost its replication by regulating the host's energy and amino acid metabolism in the early stages of infection [23]. Consequently, the integration of multiple omics analyses can reveal molecular variations across different dimensions, facilitating a comprehensive understanding of the intricate interactions between viruses and their hosts.

Animal challenge models play a critical role in evaluating vaccine/drug efficacy against ASFV and investigating ASFV-host interactions. However, most current studies utilize intramuscular injection-based challenge models, which induce an accelerated disease progression. This rapid pathogenesis leads to earlier and more pronounced viremia onset, potentially masking therapeutic benefits during drug development due to overwhelming viral loads before test compounds reach effective concentrations. Moreover, ASFV infection experiments mandate Animal Biosafety Level 3 (ABSL-3) containment facilities. For small-scale ABSL-3 laboratories, the physical size requirements of standard swine models create significant spatial constraints. Therefore, this study established an oral infection model of ASF in weaned Landrace piglets and comprehensively evaluated viral proliferation and pathological changes across multiple organs. ASFV primarily infects mononuclear phagocytes (e.g., macrophages), with the spleen serving as a major reservoir harboring abundant target cells [24]. Herein, transcriptomics and metabolomics were employed to analyze gene regulation and metabolic alterations in the spleen following ASF infection. Additionally, we identified several host metabolites with potential antiviral properties from those significantly altered in the infected spleens. This research enhances our understanding of the complex interactions between the ASFV and its host while offering a novel perspective for the development of innovative therapies against ASFV.

## 2. Materials and Methods

### 2.1. Cells, Viruses, and Animals

Primary porcine alveolar macrophages (PAMs) and porcine primary bone marrow-derived macrophages (BMDMs) were obtained from pigs using methods previously published in the literature [25, 26]. All cells were cultured in RPMI 1640 culture medium (Sigma–Aldrich, St. Louis, MO, USA) under standard conditions (37°C and 5% CO_2_), with the medium containing 10% fetal bovine serum (Vazyme Biotech, Nanjing, China), 100 IU/mL penicillin, and 100 µg/mL streptomycin (Meilun Biotechnology, Dalian, China). ASFV SY-1 (GenBank Login ID: OM161110) strains were propagated in both PAM and BMDM for amplification and stored at -80°C until use. Weaned piglets tested negative for ASFV antibodies and antigens.

### 2.2. Haemadsorption Assay

The HAD_50_ assay was utilized to quantify ASFV titers in both cell culture supernatants and tissue homogenates. PAMs were inoculated into a 96-well plate, and the viral samples to be tested were serially diluted tenfold from 10 to 10^−11^ using serum-free RPMI 1640 medium. These dilutions were sequentially inoculated into a 96-well plate with eight cells at each concentration. Further, the plate was placed in a 37°C, 5% CO_2_ incubator. After 24 h, 30 μL of 1% porcine red blood cells were added to each well, and the rosette phenomenon was assessed daily. The viral HAD_50_ was determined using the Reed-Muench method after recording the numbers of wells exhibiting rosettes at 7 days postinfection.

### 2.3. Animal Experiments

Animal experiments used 28-day-old weaned Landrace piglets that tested negative for PCV, PRRSV, PRV, and CSFV. Weaned piglets underwent random allocation into different groups, and were subsequently housed in negative-pressure isolation units within the Biosafety Level 3 facility of Huazhong Agricultural University, where each experiment was conducted. The piglets were assigned to an ASFV-infected group and noninfected group. The pigs of the infection group were orally inoculated with 500 HAD_50_ ASFV. Each pig was monitored daily for symptoms, such as fever, skin congestion, vomiting, diarrhea, food consumption, and cough. From day 0 to day 14 of the infection, samples were collected from the blood, pharynx, nose, and anus, and the viral titers were measured daily. Pigs that died within 8 days of infection were dissected immediately postmortem, with organs photographed to observe pathological changes, and samples were preserved for histopathology and immunohistochemistry (IHC). The surviving pigs were euthanised on day 14. The viral load of the samples was determined using quantitative PCR and HAD_50_ assay. In a parallel experiment, spleen samples from pigs at 8 days postinfection were collected for transcriptomic and metabolomic analysis.

### 2.4. Histopathology and IHC

Histopathological observations were performed using conventional hematoxylin and eosin staining. Tissue specimens were harvested from weaned large white piglets in both infected and uninfected cohorts, fixed in 4% neutral-buffered formalin (Servicebio Technology, Wuhan, China), paraffin-embedded, and sectioned into 4 μm thick slices. The tissue sections were subsequently subjected to two consecutive 20 min xylene washes, followed by dehydration through a graded ethanol series comprising two 5 min treatments in absolute ethanol, a 5 min incubation in 75% ethanol, and a final 1 min rinse with sterile distilled water. The sections were treated with hematoxylin (Solarbio Life Sciences, Beijing, China) for a duration of 5 min, and subsequently rinsed with sterile water for 1 min. Afterward, the slices underwent dehydration using 85%–95% anhydrous ethanol for 5 min, were stained with eosin (Solarbio Life Sciences, Beijing, China) for another 5 min, and were washed once more with sterile water for 1 min. Finally, the dehydrated slices were soaked in anhydrous ethanol for 10 min before being sealed with neutral gum.

IHC was conducted following established protocols described in prior methodology [27]. Samples of tissue from both infected and uninfected groups of weaned white pigs were preserved in a buffered formalin solution at a concentration of 4%, subsequently embedded in paraffin, and cut into sections measuring 4 µm in thickness. Following hydration, the tissue slices were incubated in a 0.1% trypsin solution containing 3 M calcium chloride dihydrate at 37°C for 20 min. Then, the glass slides were subsequently exposed to a monoclonal mouse antibody (1:1000 dilution, developed in our laboratory) targeting ASFV p72 protein, followed by 2 h incubation at 37°C. After incubation, the slides underwent three consecutive 5 min washes using Tris-buffered saline with Tween 20 (TBST) buffer. Subsequently, the slices were incubated with horseradish peroxidase (HRP)-conjugated goat antimouse IgG (H + L) (Abcam plc, Cambridge, UK, cat# ab6789) and washed three additional times with TBST for 5 min each. The sections were stained with a diaminobenzidine chromogenic solution for 5 min, and then washed three times with TBST (5 min per wash).

### 2.5. Quantification of Viral Load by qPCR

To quantify ASFV genomic DNA copy numbers in diverse biological specimens (including swabs, cellular supernatants, tissue homogenates, and EDTA-anticoagulated whole blood samples), the commercial FastPure Viral DNA/RNA Mini Kit was employed for nucleic acid extraction (Vazyme, Nanjing, China). The qPCR reaction comprised 0.2 pmol/µL of sense primer (5´-CTGCTCATGGTATCAATCTTATCGA-3´), 0.2 pmol/µL of antisense primer (5´-GATACCACAAGATCAGCCGT-3´), and 0.2 pmol/µL of probe (5´-FAM-CCACGGGAGGAATACCAACCCAGTG-3´-BHQ1), supplemented with 10 µL of PerfectStart II Probe qPCR SuperMix UDG (TransGen Biotech, Beijing, China), 2 µL of template DNA, and sufficient nuclease-free water to achieve a final reaction volume of 20 µL. Quantitative real-time PCR analysis was performed on an QuantStudio 6 system (Applied Biosystems, Thermo Fisher Scientific, Waltham, MA, USA) under the following amplification conditions: 50°C for 2 min for one cycle; 94°C for 5 min for one cycle; followed by 40 cycles of 94°C for 5 s and 58°C for 30 s.

### 2.6. Transcriptomic Analysis

#### 2.6.1. RNA Extraction, Library Preparation, and Illumina Hiseq X Ten Sequencing

Total TRIzol (Invitrogen) was used to extract total RNA from the spleen tissue of weaned piglets according to the manufacturer's protocol. A Nanodrop 2000 was used to detect the concentration and purity of the extracted RNA, and an Agilent 2100 was used to determine the RIN value. To construct a sequencing library, the sample RNA had to meet several criteria: a total quantity of at least 1 μg, a concentration of no less than 35 ng/μL, an OD260/280 ratio of 1.8 or greater, and an OD260/230 ratio of at least 1.0. The 3' end of eukaryotic mRNA had a poly A tail structure. Using magnetic beads with Oligo (dT) and poly-A to perform AT base pairing, mRNA was isolated from the total RNA for transcriptome analysis. Fragmentation buffer was added, appropriate conditions were selected, and the mRNA was randomly fragmented to ~300 bp. Under the action of reverse transcriptase, random primers were used to reverse synthesize single-stranded cDNA using mRNA as a template, followed by double-stranded synthesis. The double-stranded cDNA structure featured overhanging sticky ends. To convert it into a blunt-ended configuration, an End Repair Mix was applied. Subsequently, a single A nucleotide was incorporated at the 3' end to facilitate attachment of the Y-shaped adapter. Following adapter ligation, the resulting products were purified, and the fragments were size-selected for further processing. These fragments underwent PCR amplification and subsequent purification steps, ultimately yielding the finalized library. QuantiFluor dsDNA System quantification (Promega Corporation, Madison, WI, USA) was used, with a mix according to the data ratio. Cluster generation was achieved through Bridge PCR amplification on the cBot, and subsequent sequencing was carried out on an Illumina NovaSeq 6000.

#### 2.6.2. Differential Gene and Bioinformatics Analysis

To understand the differentially expressed genes (DEGs) between samples, Cufflinks software (http://cole-trapnelllab.github.io/cufflinks/) was used based on the existing reference genome [28]. StringTie (http://ccb.jhu.edu/software/stringtie/) was used to obtain genes without annotation information, and potential new genes were functionally annotated [29]. Using RSEM, the read counts of each sample gene were obtained using the results of alignment to the genome and the genome annotation file. The TPM conversion was performed to obtain normalized gene expression levels. Subsequently, gene expression difference analysis between samples was performed on multi-sample (≥2) projects to identify DEGs between samples, and then their functions were studied. DEGs were statistically analyzed using DESeq2, DEGseq, and edgeR software. Additionally, Gene Ontology (GO) annotation and Kyoto Encyclopedia of Genes and Genomes (KEGG) functional enrichment analyses were performed using Goatools (https://github.com/tanghaibao/GOatools) and R (http://bioconductor.org/packages/stats/bioc/edgeR), respectively.

### 2.7. Metabolomic Analysis

#### 2.7.1. Sample Extraction and QCs Sample Preparation

Frozen spleen tissue samples were retrieved from the −80°C refrigerator and thawed. Subsequently, a 2 mL centrifuge tube was prepared by adding 50 mg of the sample along with a grinding bead measuring 6 mm in diameter. Further, 400 µL extraction solution (methanol:acetonitrile = 1:1) was added to the sample, containing 0.02 mg/mL internal standard (L-2-chlorophenylalanine). This mixture was ground with a frozen tissue grinder for 6 min (-10°C, 50 Hz). Cryogenic ultrasound was employed to extract the samples for 30 min at 5°C and 40 KHz. Afterwards, the samples were allowed to sit at −20°C for an additional 30 min, after which they were centrifuged at 12,000 rpm and 4°C for 15 min. The supernatant (20 µL) was pipetted into an injection vial with an internal cannula for analysis, mixed, and used as a quality control sample (QC). In the course of instrument analysis, a QC was included after every 5–15 analyzed samples to assess the stability of the overall detection procedure.

#### 2.7.2. Data Preprocessing and Statistical Analysis

All metabolites identified based on mass spectrometry were compared with the KEGG and HMDB databases to obtain annotations of the metabolites in the databases and perform statistical analyses. Bioinformatics analysis was performed on the Masbio Cloud platform (https://cloud.majorbio.com) using the ropls R software package. Principal-component analysis (PCA) analysis and OPLS-DA have been used to describe metabolic changes in both groups and have different meanings (VIP >1.0) and combined with fatal tests (*p* < 0.05). The KEGG pathway was enriched using ropls (R) and scipy (Python). Comprehensive transcriptome and metabolome analyses using iPath 3.0 were used to identify significantly altered metabolic pathways.

### 2.8. Cytotoxicity Assay

To assess the toxicity of the metabolic compounds in cells, a CCK-8 kit (GlpBio, Montclair, CA, USA) was used to evaluate cell viability. PAMs cells were plated in 96-well plates followed by addition of small molecule compounds at 10 μmol/L final concentration. The cells were treated with 10 μM metabolites. Following 48 h incubation, 10 μL CCK-8 solution was introduced into each well prior to 2 h dark-phase incubation at 37°C. The OD_450_ was measured using a Bio-Tek microplate reader (BioTek Instruments, Winooski, VT, USA). Cellular viability quantification was performed following the manufacturer's established protocol.

### 2.9. Effect of Metabolites on ASFV Proliferation

To further investigate the effect of splenic metabolites (MedChemExpress, MCE, Monmouth Junction, NJ, USA) on ASFV replication, PAMs were seeded in 24-well plates at a density of 2 × 10^5^ cells per well. PAMs were infected with ASFV at an MOI of 0.1 and incubated at 37°C for 2 h to facilitate viral attachment. After removing unbound virions through three PBS washes, the cells were maintained in complete medium containing 10 µM small molecule compounds for 72 h. The supernatant was harvested and assessed via qPCR. To determine metabolite inhibition efficacy, ASFV genomic DNA levels in small molecule-treated PAMs were quantified and compared with those from vehicle control samples.

### 2.10. Western Blotting Analysis

To conduct the western blot analysis, the cellular proteins were initially separated using SDS–PAGE, and subsequently, they were transferred to a nitrocellulose (NC) membrane (GE Healthcare Life Sciences, Chicago, IL, USA) utilizing a transfer buffer. Blocking of the membrane was performed with 1% BSA for 1 h at 37°C. Following this, the membrane was treated with an ASFV p30 monoclonal antibody, which was developed in our laboratory, along with a rabbit antiGAPDH antibody (1:3000 dilution, Cali-Bio, Coachella, CA, USA) for 2 h at 37°C. Afterward, the membrane was washed three times using TBST. For the final step, incubation of the membrane with a HRP-conjugated AffiniPure goat antimouse IgG (H1L) secondary antibody (diluted 1:5000; Proteintech, Wuhan, China) was conducted at 37°C for 1 h, followed by three additional washes with TBST. The specific bands on the membrane were then detected using the western blotting ECL reagent (Advansta, San Jose, CA, USA).

### 2.11. Statistical Analysis

For transcriptomics analysis, the threshold for DEGs was set at |log2FC|> 1 and *p* adjust < 0.05. GO enrichment analysis and KEGG pathway analysis were performed using cluster Profiler (version 3.12.0). For metabolomic analysis, XCMS software (https://xcmsonline.scripps.edu/index.php) was used to analyze the raw data for peak alignment, calibration, and retention time peak area extraction. SIMCA-P 14.1 software package (V14.1; Sartorius Stedim Data Analytics AB, Umea, Sweden) was used for multidimensional statistical analysis, including PCA and supervised partial least-squares discriminant analysis (PLS-DA). The PCA maps, volcano maps, and cluster maps were generated with the R program. KEGG enrichment analysis was utilized to determine the metabolic pathways. Statistical significance was assessed using unpaired Student's *t*-test and GraphPad Prism 6.0.2 software (San Diego, CA, USA). Statistical significance was defined as *⁣*^*∗*^*p*  < 0.05, *⁣*^*∗∗*^*p*  < 0.01, *⁣*^*∗∗∗*^*p*  < 0.001, and *⁣*^*∗∗∗∗*^*p*  < 0.0001.

## 3. Results

### 3.1. Replication and Shedding Dynamics of ASFV in Orally Infected Weaned Landrace Pigs

**Figure 1:**
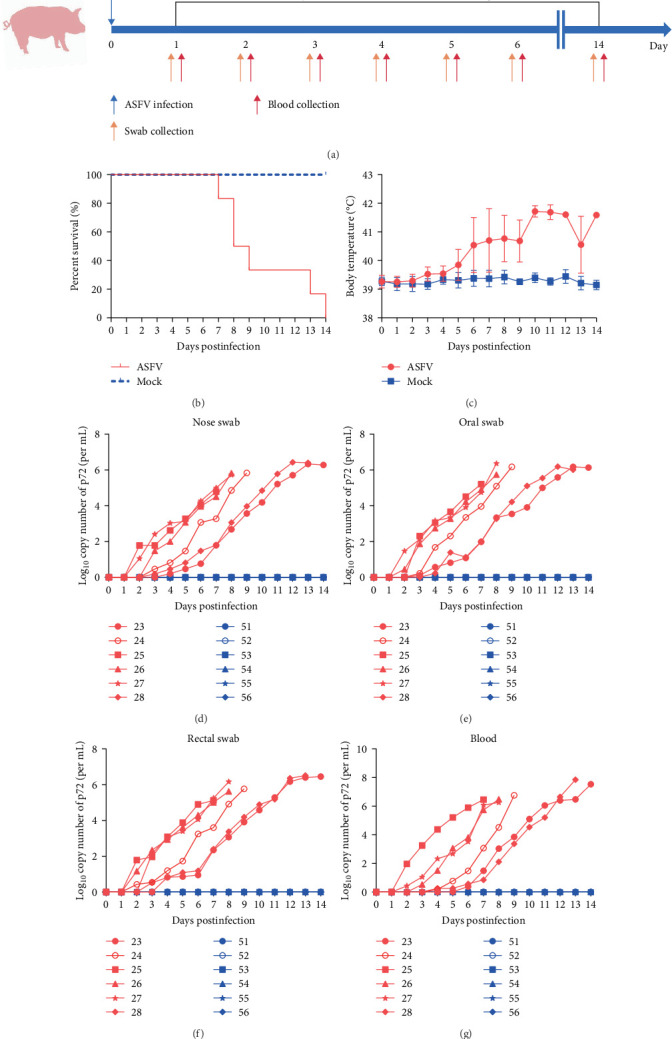
Schematic overview of the established infection model. (A) landrace pigs were infected with ASFV SY-1 doses of 500 HAD_50_. Swabs and blood were collected daily for 14 days postinfection, the body temperature changes (B), and body temperature (C) were monitored every day after infection. Daily viral genomic DNA copies were determined from nasal (D), oral (E), and rectal (F) swabs of pigs from different groups after infection. (G) Viral genomic copies in blood samples of ASFV SY-1-infected weaned Landrace pigs measured daily across experimental groups.

In our previous studies, we successfully isolated a type II ASF strain designated SY-1, which displayed a genomic profile similar to that of Pig/HLJ/18 [30]. In this study, we orally infected 28-day-old Landrace piglets with 500 HAD_50_ doses of the SY-1 strain or PBS and monitored their survival and temperature fluctuations for 14 days. Additionally, we collected blood samples and rectal swabs from these pigs on days 1, 2, 3, 4, 5, 6, and 14 postinfection (dpi) (Figure 1A). All piglets afflicted with ASFV succumbed within a span of 14 days (Figure 1B), exhibiting a consistent rise in their body temperature prior to mortality, surpassing 40°C at 6 days dpi. In contrast, the body temperature of noninfected piglets remained stable at ~39°C throughout the observation period (Figure 1C). The viral load in the nose, oral, and rectal swabs, and blood samples was quantified by qPCR, and we found that the viral genome copy number was detectable as early as 2–4 dpi across various samples, and it progressively escalated with the advancement of infection until death. Notably, peak viral copy numbers in nasal, oral, and rectal specimens reached 2–3 × 10^6^ copies/mL, while blood samples demonstrated a substantially higher maximum viral load of 7.08 × 10^7^ copies/mL (Figure 1D–G).

### 3.2. Organ Lesions and Viral Load of Infected Weaned Landrace Pigs

**Figure 2:**
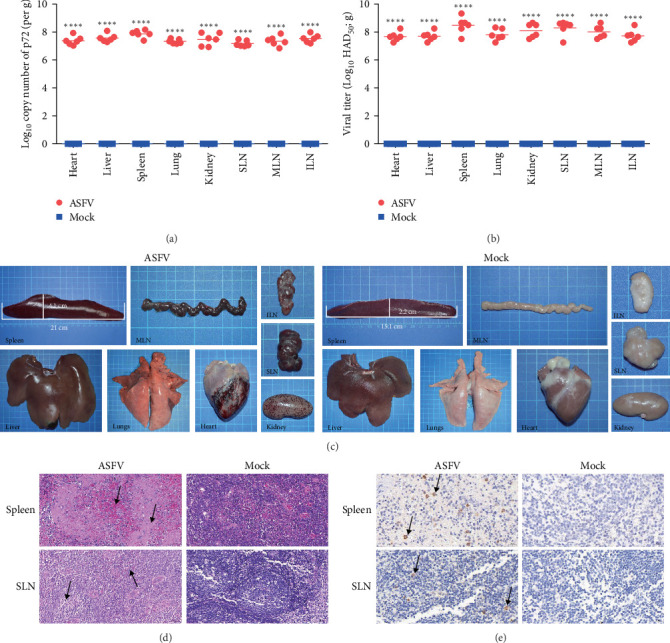
Gross lesions of different tissues of landrace pigs that died as a result of infection with African swine fever virus (ASFV) SY-1. (A) Viral genomic DNA copies and (B) viral titer in the heart, liver, spleen, lung, kidney, SLN, ILN, and MLN of pigs at death or up to 14 dpi. (C) Tissue samples, including those of the heart, liver, spleen, lung, kidney, SLN, ILN, and MLN, from weaned landrace pigs infected with ASFV SY-1 at 500 HAD_50_ that died within 8 days postinfection compared to the mock group. (D) Hematoxylin and eosin staining assay showing subtle pathological changes in the spleen and SLN of pigs. Black arrows indicate lesions in tissue samples. (E) Immunohistochemistry using antibodies to ASFV p72 proteins. ILN, inguinal lymph node; MLN, mesenteric lymph node; SLN, submaxillary lymph node. Data were analyzed using a two-tailed Student's *t*-test (*⁣*^*∗∗∗∗*^*p*  < 0.0001).

Further detection of viral genome copies and infectious viral particles in organs such as the heart, liver, spleen, lungs, and kidneys of ASFV-infected pigs was performed. The results showed that the copy number of the P72 gene in each organ was higher than 1.30 × 10^7^ copies/g, and the number of infectious virus particles per gram of tissue was as high as 4.76 × 10^7^ HAD_50_/g (Figure 2A,B). Among the various organs, the spleen had the highest viral load. Multiple organ lesions were observed in weaned piglets following oral administration of ASFV. Compared to uninfected pigs, those infected with ASFV showed bleeding in the heart, mesenteric lymph nodes (MLNs), submandibular lymph nodes (SLNs), and inguinal lymph nodes (ILNs), and needle-shaped bleeding points appeared in the kidneys. In addition, the spleen showed significant bleeding and enlargement, with an average increase in length and width of 5.9 and 1.9 cm, respectively (Figure 2C). Subsequently, the spleens and SLNs of uninfected and ASFV-infected pigs were sectioned and stained with hematoxylin and eosin for histopathological analysis. These results indicated that the fundamental structures of the spleen and SLNs in noninfected pigs were normal. Nevertheless, in ASFV-infected pigs, the interstitial tissue of the spleen was markedly congested, the boundary separating the red pulp from the white pulp was not clearly defined, the spleen cells were necrotic, and a significant number of red blood cells and lymphocytic infiltration was observed between the tissues. A minor amount of inflammatory cell exudation and ferritin deposition in the SLNs was seen (Figure 2D). IHC analysis revealed the distribution of ASFV particles in the spleen and SLNs of infected pigs (Figure 2E). These results indicated that ASFV infection in weaned white pigs led to severe organ pathology, with the spleen being the most severely affected organ.

### 3.3. Effects of ASFV Infection on the Spleen Transcriptome

**Figure 3:**
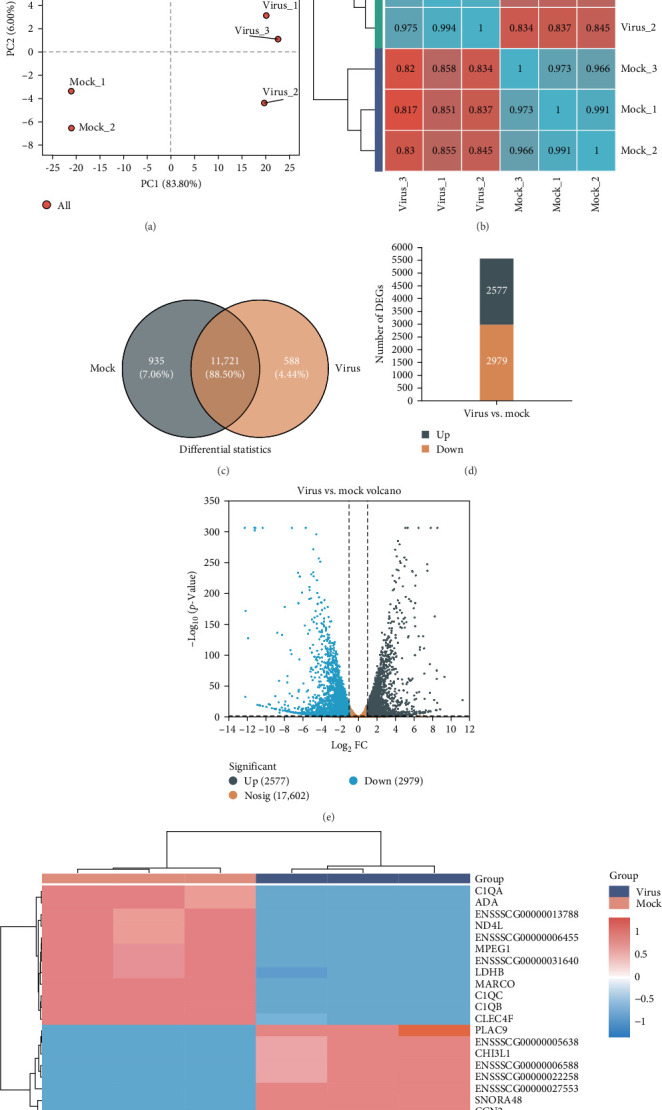
The effect of ASFV infection on spleen transcriptome. The weaned landrace was infected with a dose of 500 HAD_50_ of the ASFV SY-1 strain, and spleens from both infected and noninfected pigs were collected on day 8 postinfection for transcriptomic analysis. (A) The principal component analysis (PCA). (B) The degree of variation in gene regulation between samples is quantitatively analyzed through correlation data between samples. Each grid in the figure represents the correlation between two samples, different colors represent the relative size of the correlation coefficient between samples, and the length of the clustered branches represents the relative distance between samples. (C) The Venn diagrams show the shared genes in the two groups. (D) The upregulated, downregulated, and the total number of DEGs at 8 day after landrace pigs infected by ASFV. (E) Volcano plots for DEGs between virus and mock groups of pigs. The dotted line across the volcano plots shows the cutoff for DEGs >95% confidence (*p*  < 0.05). (F) Heatmap analysis of DEGs in spleen at 8 day postASFV infection. Gene expression level is indicated using a color key, where blue corresponds to low-level and red corresponds to high-level expression.

The spleens of infected and uninfected pigs were subjected to RNA-seq analysis to identify the regulation of host genes following ASFV infection. After data processing, a total of 50,333,549 and 42,549,380 reads were generated from the mock and virus, respectively, representing a total of 7.34 and 6.24 Gb nucleotides. The Q30 content, referring to sequences with sequencing error rates below 0.1%, for both cDNA libraries surpassed 93.93%, while the GC composition in each sample was greater than 50.46% (Table 1). PCA showed that intergroup samples were scattered and intragroup samples were clustered, suggesting good intragroup duplication and significant intergroup differences (Figure 3A). The Pearson correlation confirmed PCA findings, with robust concordance among biological replicates. (Figure 3B). A total of 13,244 genes were identified in the viral and mock groups, with 11,721 genes shared by both groups (Figure 3C). Through differential expression analysis, a total of 5556 genes with significant differences were identified in the virus-treated group compared to the mock control group (|log2FC|> 1 and *p* adjust < 0.05). Among these, 2577 genes were found to be upregulated, while 2979 genes were downregulated (Supporting Information 1: Table S2), which are shown with bar graphs and a volcano plot (Figure 3D,E). We further screened the top 20 significantly upregulated and downregulated genes between the mock and virus groups and displayed the results using heatmaps. These findings indicate that PLAC9, CHI3L1, SNORA48, and CCN2 were significantly upregulated in the spleen following infection, whereas C1QA, ADA, ND4L, MPEG1, LDHB, MARCO, C1QC, C1QB, and CLEC4F were significantly downregulated (Figures 3F).

**Figure 4:**
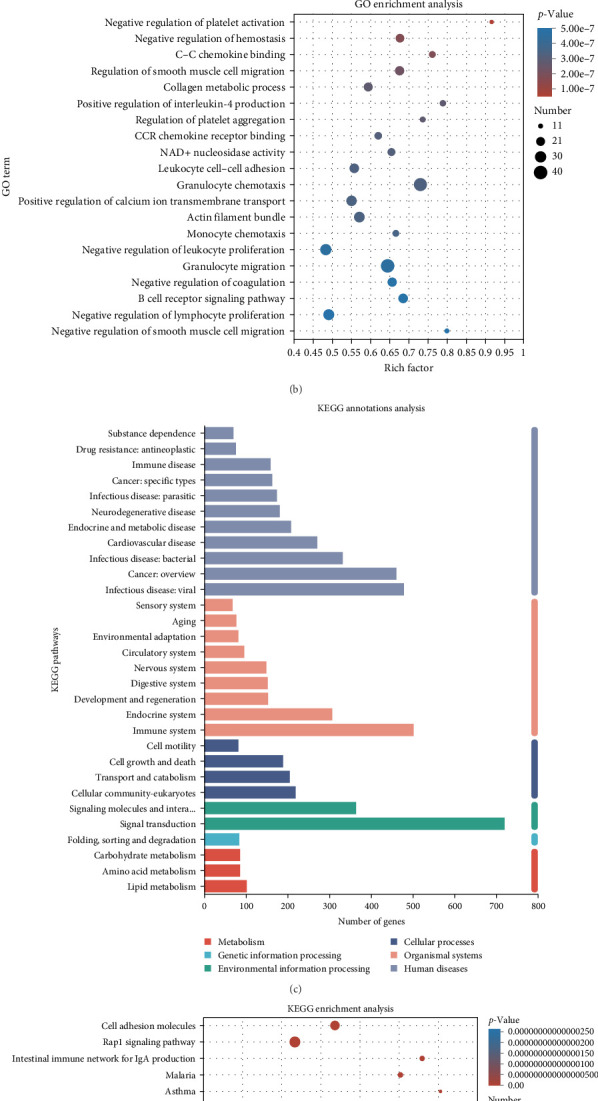
The results of GO and KEGG pathway enrichment of DEGs. (A) GO annotations analysis were clustered into three pathway categories: biological process, cellular component, and molecular function. (B) The top 20 GO enrichment analysis of DEGs between mock and virus groups. (C) The significantly enriched KEGG pathways were clustered into six pathway categories: metabolism, environmental information processing, organismal systems, genetic information processing, cellular processes, and human diseases. (D) The top 20 KEGG enrichment analysis of DEGs between mock and virus groups.

To elucidate their potential functions and metabolic pathways, GO and KEGG annotations were employed for further analysis of DEGs. Our findings indicate that gene regulation following ASFV infection primarily pertains to the immune system process, multicellular organismal process, localization, cellular component organization or biogenesis, developmental process, response to stimulus metabolic, process biological regulation and cellular process. In the cellular component category, most of DEGs were associated with cell parts, organelles, and membranes. The molecular function category was enriched for molecular transducer activity, molecular function regulators, catalytic activity, and binding (Figure 4A). GO enrichment analysis revealed that the DEGs were primarily clustered in the negative regulation of platelet activation, negative regulation of haemostasis, C–C chemokine binding, negative regulation of smooth muscle cell migration, collagen metabolic processes, and positive regulation of interleukin-4 production, etc. (Figure 4B). The top six KEGG terms identified were “organismal systems,” “human diseases,” “cellular processes,” “metabolism,” “environmental information processing,” and “genetic information processing,” and the immune system, signal transduction and infectious diseases (viral) related pathways, contained the largest number of genes (Figure 4C). Furthermore, KEGG pathway enrichment analysis revealed that the DEGs were predominantly enriched in cell adhesion molecules, the Rap1 signaling pathway, cytokine–cytokine receptor interactions, and the Ras signaling pathway (Figure 4D).

### 3.4. Impact of ASFV Infection on the Spleen Metabolic Profile

**Figure 5:**
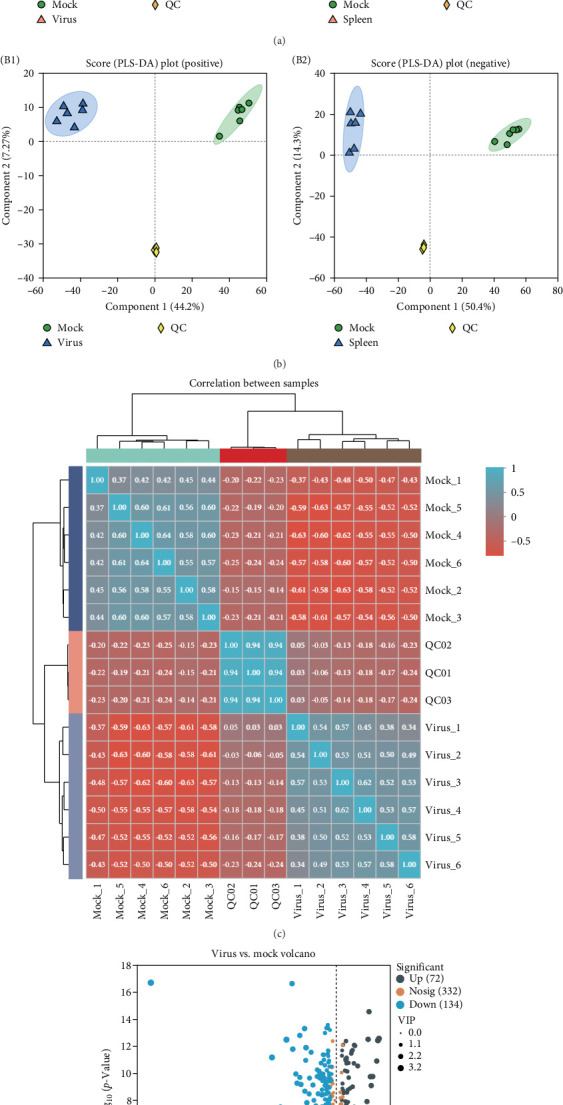
Significant differential metabolite levels in pig spleens after ASFV infection. The weaned landrace was infected with a dose of 500 HAD_50_ of the ASFV SY-1 strain, and spleens from both infected and noninfected pigs were collected on day 8 postinfection for metabolomics analysis. (A) The PCA of the sample in mock and virus group. A1 represents the positive ion mode, and A2 represents the negative ion mode. (B) OPLS-DA showing difterences in metabolite composition between the mock and virus group. B1 represents the positive ion mode, and B2 represents the negative ion mode. (C) The cluster heatmap shows the correlation between samples. (D) Volcano plot comparison of the mock and virus group. Each point in the volcano plot represents a metabolite, the abscissa represents the fold change of each substance in the group compared to each other (taking the logarithm with base 2), and the ordinate represents the *p*-value of the Student's *t*-test (taking the pair with base 10).

Metabolic changes in the spleens of uninfected and ASFV-infected piglets were evaluated using UPLC–MS/MS. PCA revealed significant differences between the mock and virus groups in both the positive and negative models (Figure 5A). Moreover, OPLS-DA was employed to examine both nonorthogonal and orthogonal variables in order to gather more dependable data on metabolic alterations following ASFV infection, with the first principal component explaining 44.2% of the total variance in the comparison of mock vs. virus (Figure 5B). The degree of variation in metabolite composition and abundance among the samples was quantitatively assessed using correlation data, which indicated strong sample repeatability within each group (Figure 5C). Analysis of untargeted metabolomics indicated that ASFV infection caused significant alterations in the levels of 206 metabolites; specifically, 134 metabolites showed a notable decrease, while 72 metabolites exhibited a significant increase (Figure 5D and Supporting Information 2: Table S3).

**Figure 6:**
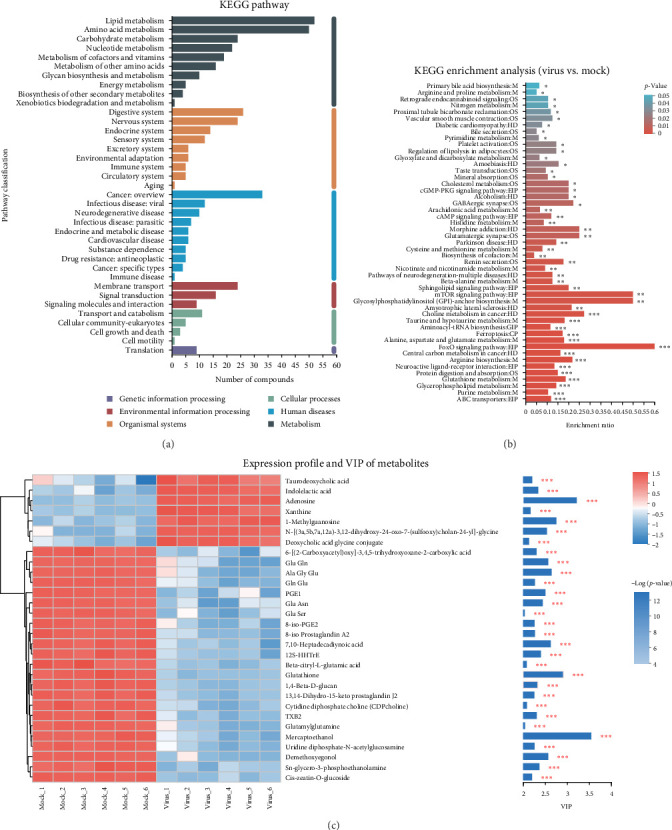
The differential metabolite levels and metabolic pathways in pig spleens after ASFV infection. (A) KEGG Pathway and (B) enrichment analysis of differential metabolites between mock and virus group. (C) Top30-fold change bar chart, exhibited the top10 most significantly upregulated or decreased metabolites. Red represented increased metabolites and green represented decreased metabolites. *⁣*^*∗*^*p*  < 0.05, *⁣*^*∗∗*^*p*  < 0.01, *⁣*^*∗∗∗*^*p*  < 0.001.

Differential metabolites postASFV infection were mapped to associated metabolic pathways using the KEGG metabolome database. (Figure 6A). Particularly, KEGG enrichment analysis suggested several enriched metabolites, including those of the FoxO signaling pathway, glycosylphosphatidylinositol (GPI) synthesis, choline metabolism in cancer, and the mTOR signaling pathway (Figure 6B). The top 30-fold change distribution plot and bar chart showed seven upregulated metabolites in ASFV including taurodeoxycholic acid, indolelactic acid, adenosine, xanthine, 1-methylguanosine, N-([3a, 5b, 7a, and 12a]−3,12-dihydroxy-24-oxo-7-[sulfooxy] cholan-24-yl)-glycine, deoxycholic acid glycine conjugate, and 27 downregulated metabolites in ASFV, including Glu, Gln, Ala, Gly, Glu, PGE1, Glu, Asn, 7,10-heptadecadiynoic acid, glutathione, 1,4-beta-D-glucan, mercaptoethanol, demethoxyegonol, and Sn-glycero-3-phosphoethanolamine (Figure 6C).

### 3.5. Integrated Analysis of Transcriptome and Metabolome in the Spleen After ASFV Infection

**Figure 7:**
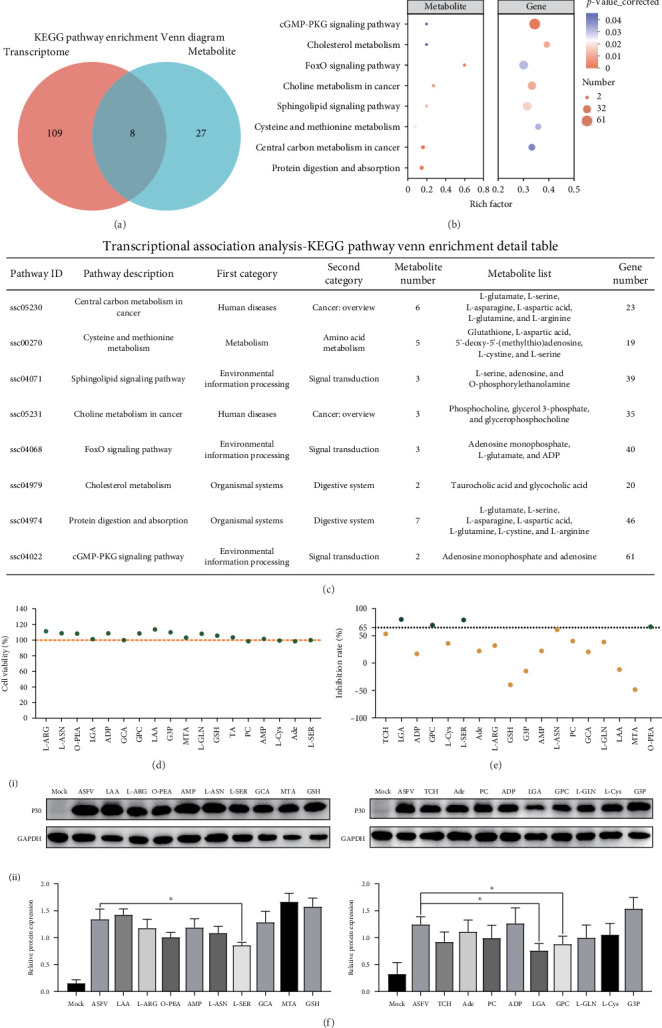
(A) The Venn diagram shows the overlap and differences in KEGG enrichment pathways between transcriptome and metabolome. (B) The bubble plot and table and (C) display the overlapping KEGG enrichment pathways and related metabolite information between the transcriptome and metabolome. (D) PAMs cells were treated with 10 μM metabolites for 24 h. Viability was determined using CCK-8 assays. (E) The effect of metabolites on ASFV proliferation was detected by qPCR, and the inhibition rate was calculated. (F)The function of the metabolites assessed using western blot analysis. (i) ASFV p72 and porcine glyceraldehyde-3-phosphate dehydrogenase (GAPDH) proteins were detected. (ii) The density of the ASFV p72 protein bands was analyzed using Image-J software and normalized to GAPDH. Data were analyzed using a two-tailed Student's *t*-test (*⁣*^*∗*^*p*  < 0.05).

In order to explore the association between differential metabolites and DEGs in the spleens of the mock and virus groups, the integration of KEGG pathway enrichment results was carried out. The Venn diagram analysis revealed coenrichment of differential metabolites and DEGs in eight KEGG pathways, including cysteine and methionine metabolism, cGMP-PKG signaling, choline metabolism in cancer, cholesterol metabolism, sphingolipid signaling, protein digestion and absorption, FoxO signaling, and central carbon metabolism in cancer (Figure 7A,B). We identified 18 metabolites, including L-aspartic acid and L-glutamine, based on these eight metabolic pathways (Figure 7C). Initially, we assessed the cytotoxic effects of 18 metabolites and found that a concentration of 10 μM did not significantly impact the viability of PAMs. (Figure 7D). Additionally, we assessed the impact of these small molecules on ASFV infection in PAMs. The ASFV P72 gene copy number and p30 protein expression levels were quantified by qPCR and western blot analysis, respectively. The results indicated that 10 μM L-glutamate, glycerophosphocholine, and L-serine significantly inhibited ASFV proliferation in PAMs (Figure 7E,F).

## 4. Discussion

ASF and its hosts have undergone coevolution for over a century, and some strains have been studied for decades. Owing to the lack of vaccines or treatment methods, current measures to deal with ASF still mainly rely on biosecurity prevention and control. The establishment of experimental animal challenge models will deepen our understanding of the interaction between viruses and hosts and promote the development of ASFV prevention and treatment approaches. Most current vaccine efficacy evaluations in challenging experiments use intramuscular injections. When pigs are infected with the Georgia strain at a dose of 10^3^–10^4^ HAD_50_, all infected pigs must be euthanized within 5–6 days[31], whereas pigs infected with 10^2^ HAD_50_ ASFV require euthanasia within 6–7 dpi [32]. A previous study investigated the pathogenicity of different doses of ASFV following intramuscular injection in Panamanian pigs. The results indicated that all pigs infected with ASFV SY-1 at doses exceeding 10^2^ HAD_50_ succumbed within 7 days, with detoxification detected between the 2nd and 4th day postchallenge. Notably, the time to death in infected pigs was significantly delayed only when the infection dose was below 10 HAD_50_ [30]. However, Bama Xiang pigs infected orally with 500 HAD_50_ began detoxification 3–4 dpi and did not succumb until 17 dpi [33]. This observation indicates that the pathological reactions in pigs infected via intramuscular injection are more acute than those in pigs infected through oral administration, requiring significantly lower doses to markedly delay disease progression. The transmission of AFSV mainly occurs through the consumption of contaminated feed ingredients or direct contact with infected pig herds [34]. The ingested ASFV particles initially replicate in the macrophages of the tonsils or superficial lymph nodes before dissemination throughout the body via the bloodstream and lymphatic system [35]. Consequently, the oral challenge is more representative of the natural transmission dynamics of ASFV among pig populations, suggesting that it may serve as a more appropriate in vivo model for research related to ASFV vaccines or therapeutics. Moreover, compared to pigs infected via intramuscular injection, pigs infected with the ASFV through oral transmission exhibit a slower progression of illness, which may facilitate understanding of ASFV-host interactions, encompassing detoxification trends, infection pathology, metabolic changes, and gene regulation. However, compared to the intramuscular injection challenge method, the oral challenge approach exhibits greater susceptibility to operational variables and environmental influences, which may be its limitation. This study established an oral ASF infection model in Landrace pigs using a dose of 500 HAD_50_. All pigs were euthanised 14 dpi, which was earlier than the duration required to euthanize Panama pigs orally infected with 500 HAD_50_ [33]. These findings suggest that Landrace pigs exhibit greater susceptibility to ASFV than Bama minipigs. Furthermore, the age of the experimental animals is another critical factor influencing the pathological symptoms associated with ASFV infection [36]. Therefore, it is essential to select an appropriate animal model based on the specific requirements of the experiment prior to initiation.

Investigating the pathogenic mechanisms of ASFV and its regulation of the host immune response is crucial for developing effective prevention and control technologies. In this study, we examined the effects of oral ASFV infection on gene regulation and metabolic alterations in the pig spleen through transcriptomics and metabolomics, thereby enhancing our understanding of viral–host interactions. ASFV must manipulate the gene expression of host cells to sustain efficient viral replication, while simultaneously overcoming the host's innate antiviral responses to facilitate virus transmission and spread. During the early stages of ASFV infection, host organs initiate a coordinated immune response by activating immune-related genes, triggering a proinflammatory cytokine storm, and inducing the activation of interferon pathways [17]. It is noteworthy that the PI3K-AKT signaling pathway exhibited significant alterations across all examined organs (including the heart, ILN, kidneys, liver, lungs, MLN, muscles, spleen, SLN, and tonsils), which is similar to our previous findings in the Bama minipigs infection model [30], indicating that ASFV may regulate the PI3K-AKT signaling transduction pathway to evade the host's immune defense mechanisms. Previous studies conducting transcriptomic and proteomic analyses of lungs, spleens, livers, kidneys, and lymph nodes from naturally ASFV-infected pigs under field conditions have confirmed that immune system responses, complement and coagulation cascades, and metabolic processes are key pathways during ASFV infection [37]. The transcriptomic results from this study revealed significant enrichment of immune-related, metabolic, and inflammatory pathways in the spleens of pigs orally infected with ASFV. These results are consistent with the suppression of host immune system activation and evasion of natural host immunity following viral infection. However, this study primarily focused on the gene regulation characteristics in the spleen during the acute disease phase following oral ASFV infection. Future longitudinal multi-timepoint monitoring of host responses across distinct infection stages (e.g., incubation period, viremic phase, and convalescence) will enable systematic elucidation of ASFV's adaptive evolution mechanisms and its dynamic interactions with host immune-metabolic networks during oral infection. We found that following ASFV infection, genes related to splenic macrophage regulation (MPEG1 and MARCO), complement component 1q (C1q), and the mitochondrial function-related gene LDHB were significantly downregulated. In contrast, PLAC9, CHI3L1, and CN2 were significantly upregulated. In antiviral immunity, cytokines induce the expression of MPEG1 to resist viral infection. Studies have demonstrated that the partial or complete loss of MPEG1 in cells and animals can increase their susceptibility to microbial infections [38]. MARCO, a glycoprotein found in the surface of certain macrophages, binds and eliminates bacteria and their toxins. As a crucial receptor, MARCO mediates the defensive functions of macrophages and is essential in innate immunity [39, 40]. This indicates that inhibition of macrophage-related functional genes by ASFV is a key mechanism for counteracting host immune responses. C1q is a protein complex involved in the classical complement pathway and is part of the innate immune system [41]. It unknown whether there is a correlation between its expression inhibition and the difficulty of humoral immunity against ASFV in exerting protective effects. DHB encodes lactate dehydrogenase B, which is crucial in glycolysis. Research has shown that LDHB inhibition similarly promotes mitochondrial autophagy and enhances CSFV proliferation [42] that ASFV may also facilitate self-replication through a comparable mechanism. CHI3L1 plays a crucial role in macrophage development, dendritic cell recruitment, and maintaining the balance between Th1 and Th2 responses [43–45]. The upregulation of CHI3L1 following ASFV infection may represent the host's intrinsic immune regulation as a reaction to the virus. Another study has indicated that CHI3L1 promotes the polarization of M2 macrophages, which contributes to immune evasion and tumor progression [46]. This suggests that ASFV has the potential to regulate CHI3L1 expression to facilitate immune escape. However, the specific role of CHI3L1 in ASFV infection requires further investigation.

ASFV infection induces metabolic reprograming in the host and facilitates self-replication by hijacking host metabolites. Conversely, certain host metabolites inhibit viral infection [47]. A deeper exploration of the effects of viral infections on host cell metabolism will enhance our understanding of the underlying pathogenic mechanisms. Here, we analyzed the differential metabolites and associated KEGG enrichment pathways in the spleen infected with ASFV using metabolomics, finding that the FoxO signaling pathway, GPI synthesis, choline metabolism in cancer, and the mTOR signaling pathway exhibit the highest enrichment factors following ASFV infection. It has been reported that the infection of PAMs cells by ASFV boosts host energy and amino acid metabolism in the initial stages of infection, thereby facilitating self-replication [23]. Our study also identified significant alterations in amino acid metabolism within the spleens of infected pigs, with notably enriched pathways, including histidine metabolism, aminoacyl tRNA biosynthesis, alanine, aspartate, and glutamate metabolism, arginine biosynthesis, protein digestion and absorption, and glutathione metabolism. Viruses rely on nucleotides supplied by host cells to complete their replication process, and nucleotide metabolism undergoes notable alterations in PAM cells infected with ASFV [23]. Our results indicated that, following infection with ASFV, metabolites associated with nucleotide metabolism, such as adenosine and guanosine, were significantly increased. These metabolites may serve as essential precursors of nucleotide synthesis, thereby facilitating viral replication. Glutathione, a crucial molecule for maintaining intracellular redox signaling, was significantly downregulated in the spleens of ASFV-infected pig. Depletion of glutathione in cells has been described as a mechanism by which certain viruses, including AIDS, influenza, and herpes simplex viruses, evade immune responses [48]. Studies have indicated that glutathione depletion may significantly influence the pathophysiology, host immune response, disease severity, and mortality associated with COVID-19, while treatment of lethally infected mice with glutathione analogs has been demonstrated to substantially alleviate pathological damage [49]. We evaluated the effect of glutathione on ASFV proliferation in vitro and found that it does not directly inhibit viral replication (Supporting Information 3: Figure S1A,B). The role of glutathione in vivo during ASFV infection remains to be further investigated.

Amino acids are the basic units of protein synthesis and serve as fundamental structural elements and energy sources that participate in various life activities, such as cell growth and differentiation, biosynthesis, signal transduction, and immune regulation. Amino acid metabolism disorders are associated with many pathological conditions, including metabolic diseases, cardiovascular diseases, immune diseases, cancer, and pathogenic infections [50]. The effects of viral infections on amino acid metabolism have been confirmed in multiple studies. For example, after NDV infection, the levels of tyrosine, threonine, isoleucine, serine, methionine, and alanine in cells were significantly upregulated [51]. Infection with the vaccinia virus (VACV) modifies the metabolism of glutamine, and a reduction in external glutamine results in a notable decline in the production of infectious virus, suggesting that glutamine plays an essential role in the replication of VACV [52]. Previous studies have demonstrated that ASFV infection increases the levels of L-arginine, aspartate, and glutamate, thereby facilitating replication [23, 53]. However, our results indicate that L-glutamate inhibits ASFV proliferation in PAMs cells. This suggests that glutamate plays a dual role in ASFV infection. We speculate that the glutamic acid concentration could be a critical factor contributing to this phenomenon, warranting careful consideration of the dosage in future applications. Numerous studies have confirmed that Glu signaling can influence the function of T cells [54], B cells [55], and macrophages via cell receptors [56]. Additionally, glutamate can be metabolized into glutamine and glutathione within cells, which serve as important metabolic intermediates that provide energy for the proliferation and functional regulation of immune cells [57, 58]. Nonetheless, further investigations are required to ascertain whether glutamate affects ASFV proliferation via the aforementioned pathways.

In conclusion, our study established an ASFV oral challenge model that has great potential in the evaluation of future vaccines or drugs. The integrated examination of the transcriptome and metabolome revealed gene regulation and metabolic changes in the spleen after ASFV infection, further confirming the beneficial role of L-glutamate, glycerophosphocholine, and L-serine in ASFV infection in vitro experiments. This study not only enriches the comprehension of the interaction between ASFV infection and host metabolic factors but also provides new ideas for developing novel ASF inhibitors.

## Figures and Tables

**Table 1:** Sequencing data statistics table.

Sample	Raw reads	Raw bases	Clean reads	Clean bases	Error rate (%)	Q20 (%)	Q30 (%)	GC content (%)
Virus_3	44,168,442	6,669,434,742	42,156,742	6,188,710,101	0.0249	97.98	94.41	50.8
Virus_2	47,590,292	7,186,134,092	45,521,766	6,664,144,202	0.0249	97.99	94.4	51.69
Virus_1	42,102,766	6,357,517,666	39,969,632	5,864,041,464	0.0255	97.74	93.93	50.46
Mock_3	47,773,248	7,213,760,448	45,411,118	6,641,567,500	0.0252	97.86	94.14	50.51
Mock_2	55,887,332	8,438,987,132	53,691,182	7,777,971,866	0.0247	98.07	94.73	52.24
Mock_1	54,639,620	8,250,582,620	51,898,346	7,597,228,255	0.025	97.95	94.39	51.7

## Data Availability

The transcriptome dataset has been deposited in the Gene Expression Omnibus (GEO) repository maintained by the National Center for Biotechnology Information (NCBI), with the associated BioProject accession identifier PRJNA1211204. The metabolomics datasets described in this study are publicly accessible through the MetaboLights repository (ID: MTBLS12175).

## Supporting Information

Supporting Information 1 Table S2. Differential gene between Mock group and Virus group.

Supporting Information 2 Table S3. Differential metabolites between Mock group and Virus group.

Supporting Information 3 Figure S1. Glutathione has no effect on the replication of ASFV in vitro (Supporting figure).
