# Supplementary material for: Infection Characteristics, Transcriptomics, and Metabolomics of African Swine Fever Virus SY-1 Strain in Orally Infected Weaned Landrace Piglets
Source: Transbound Emerg Dis. 2025 Jul 14;2025:2453420. doi: 10.1155/tbed/2453420 (PMC12279419; doi:10.1155/tbed/2453420)
Supplement: Supporting Information 3 — Figure S1. Glutathione has no effect on the replication of ASFV in vitro (Supporting figure). [file 2453420.f3.docx]

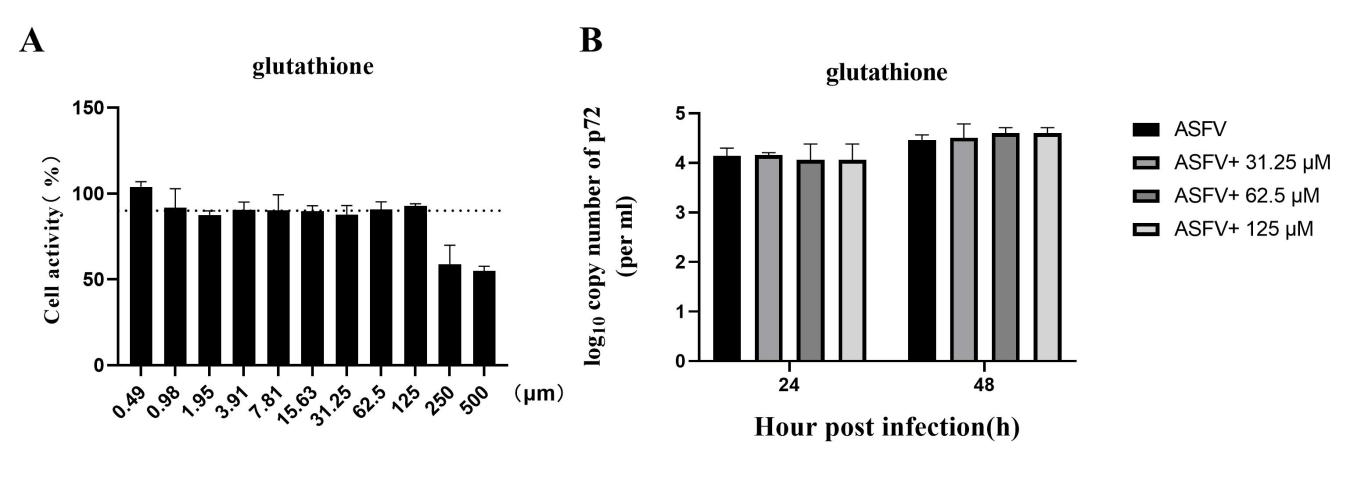


Figure S1. Glutathione has no effect on the replication of ASFV in vitro. (A) PAMs cells were treated with 31.25, 62.5 and 125μM glutathione for 24 h. Viability was determined using CCK-8 assays. (B)The effects of metabolites on the proliferation of African swine fever virus (ASFV) were detected by qPCR, and the viral copy numbers in the cell supernatants were calculated.
